# A stopped-flow/flow-injection system for automation of α_2_ macroglobulin kinetic studies

**DOI:** 10.1155/S1463924683000103

**Published:** 1983

**Authors:** C. Riley, B. F. Rocks, R. A. Sherwood, L. H. Aslett, P. R. Oldfield

**Affiliations:** 1 School of Engineering and Applied Sciences University of Sussex Brighton BN1 9QT UK; 2 Biochemistry Department Royal Sussex County Hospital Brighton BN2 5BE UK; 3 School of Molecular Sciences University of Sussex Brighton BN1 9QT UK


Journal of Automatic Chemistry, Volume 5, Number 1 (January–March 1983), pages 32–35

### A stopped-flow/flow-injection system for automation of $\alpha_2$ macroglobulin kinetic studies

**C. Riley**
*School of Engineering and Applied Sciences, University of Sussex, Brighton BN1 9QT, UK*

**B. F. Rocks, R. A. Sherwood, L. H. Aslett**
*Biochemistry Department, Royal Sussex County Hospital, Brighton BN2 5BE, UK*

**and P. R. Oldfield**
*School of Molecular Sciences, University of Sussex, Brighton BN1 9QT, UK*

### Introduction

The technique developed by Topping and Seilman [1] for the classification of $\alpha_2$ macroglobulin is mentioned in outline in the preceding paper [2]. In this, a dilutor is described which prepares the 25 dilutions of serum required for each analysis. In the second part of the analysis, a portion of each diluted serum (0·1 ml) is added to 3 ml of an artificial substrate consisting of a solution of N-$\alpha$-Benzoyl-L arginine ethyl ester (BAEE) in Tris (hydroxy methyl) methylamine (Tris) buffer and the rate of change of absorbance at 253 nm is measured. A means was needed to mechanize this second part of the analysis and the use of flow-injection in its stopped-flow/kinetic mode appeared to offer a good solution to the problem.

Flow-injection analysis (FIA) was developed more or less simultaneously in 1975 by Růžička and Hansen in Denmark [3] and by Stewart *et al.* in the USA [4]. In its earliest and simplest form, the reagent is pumped through a narrow bore (< 1 mm) tube and the sample is injected by syringe as a discrete bolus or slug into the flowing stream. Mixing with reagent takes place by radial diffusion and the reaction products are observed in a flow-through detector at some distance downstream. This simple system serves well for rapid reactions, but for slower reactions, and for full mechanization, something more sophisticated is needed. Růžička *et al.* [5], Mindegaard [6] and Anderson [7] have described rotary valves in which the cavity in the valve serves to measure the sample accurately and also to transfer it into the flowing stream of reagent. These, if motorized and used in conjunction with a sample turntable, may form the basis of a completely automatic system.

Růžička and Hansen [8] have also described the use of FIA in the stopped-flow/kinetic mode; because the assembly of tubing used in FIA is relatively inelastic, and because air segmentation is not used, once the pump is stopped the slug is arrested and will remain immobile for long periods. By arresting the slug in the appropriate detector, the progress of the reaction can be monitored and recorded.

The following describes the construction of a stopped-flow/flow-injection machine dedicated to the $\alpha_2$ macroglobulin kinetic studies described above.

### Instrumentation

The flow diagram of the system is shown in figure 1, in which the turntable T and sample probe S are embodied in a Sampler II (Technicon Instruments Company, Basingstoke, Hampshire,

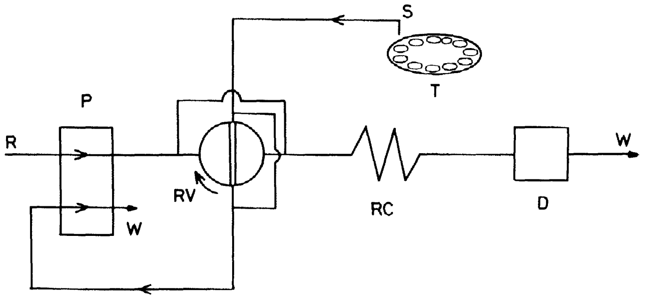

*Figure 1. FIA system. Where R = reagent, P = pump, RV = rotary valve, RC = reaction coil, D = spectrophotometer, T = automatic sample turntable, W = waste and S = sample probe.*

UK); P is a four-channel peristaltic pump (Ismatec Mini S 840, from Frost Instruments, Wokingham, Berkshire, UK). This is a fixed-speed pump driven by a synchronous motor and gives flow rates of 0·2–13 ml/min depending on the pump tubes used. RV is a rotary valve, which is a modified form of that described by Růžička and Hansen [5] and which will be described in detail later. D is the detector, a Varian 634 spectrophotometer with a heated cuvette housing (Varian Associates Ltd, Walton-on-Thames, Surrey, UK). Signals from this are recorded on a Linseis LY 1800 recorder (Linseis GmbH, Grays, Essex, UK). The cuvette is by Hellma, Model 178.13 (Hellma [England] Ltd, Westcliff-on-Sea, Essex, UK); this is made of quartz, and has a light-path of 1 mm diameter and 10 mm long. The cuvette housing is fed with water at 30°C from a water-bath, which also circulates water to baths containing the reaction coil and the substrate reservoir.

The pipeline arrangements are straightforward: the probe S is of 0·3 mm bore stainless-steel tubing connected to the valve by 1 mm bore PVC tubing. Pump tubes are of Technicon 'Tygon' PVC and the reaction coils RC are of PTFE. The sizes of the pump tubes and reaction coils are varied with the experimental conditions. The bypasses around the valve consist of 100 mm lengths of 0·3 mm bore PTFE tubing. The connections were made by sleeving the joints with sections of pump tubing of appropriate sizes, care being taken to butt the ends of the pipeline as closely as possible.

### *The rotary valve*

Since difficulties were encountered in making this operate effectively it is discussed here in some detail. It is shown in exploded form in figure 2. The central rotary part of the valve (the barrel, B) is made of PTFE (20 mm thick and 30 mm diameter). A vertical port, 0·8 mm diameter, is drilled in this 5 mm from the edge. The upper and lower plates are of 10 mm

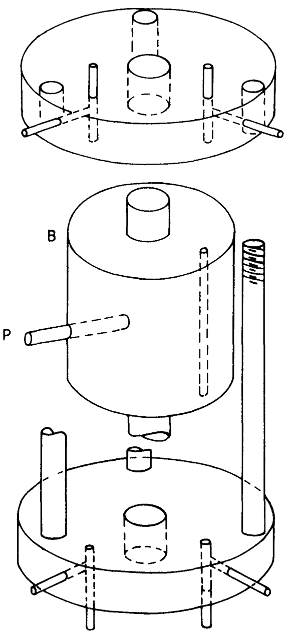

*Figure 2. Exploded view of rotary valve. Where B = barrel and P = pin.*

thick perspex and these have two vertical ports, also 0·8 mm diameter, located so that they are exactly aligned with the port in the barrel at each end of its arc of travel. The pin P passes through the barrel and is threaded into the centre shaft. It thus serves to locate the barrel on the centre shaft; it also serves as a handle to move the barrel manually and as a stop, locating the barrel positively when it comes into contact with one or other of the supporting pillars. The three supporting pillars are made of stainless-steel; they are pinned to the lower plate and pass freely through the holes in the upper plate. The latter is secured by light springs and nuts on the end of each pillar. Not shown in the sketch, the pillars extend below the lower plate to support the valve on a 4 mm thick aluminium plate beneath which is mounted a geared reversing synchronous motor (P531 SPIII 30 RPM, McLennon Servo Supplies Ltd, Camberley, Surrey, UK). The gearbox shaft of this motor is direct-coupled to the centre shaft of the valve. In practice, the motor drives the barrel to the limits of travel of pin P and is then stalled briefly until the power is switched off.

Difficulty was encountered in making the mating surfaces of the valve completely watertight, apparently due to two reasons. Firstly, it was difficult to avoid tool marks in the machined surfaces of the PTFE and after some trial-and-error, the ends of the barrel were finally finished by gently rubbing them on fine wet and dry abrasive paper, on a glass plate, liberally lubricated with soap and water. Secondly, the alignment of the perspex end-plates could not be achieved without unduly loading the retaining springs. This was overcome by enlarging the central holes in the end plates and the barrel to give a clearance of about 0·25 mm so that the central shaft was able to float slightly.

The cycle is initiated by the Technicon Sampler II which was modified as follows. The lands of a 30 sample/h 1/1 sample/wash ratio cam were milled away so that only 9 mm of each was left. This gave a sample time of 20 s and a wash time of 100 s. A second microswitch was added to the one already mounted on the sample arm, and arranged to short out switch No. 1 of the cam timer (see below) each time sampling takes place.

The main control function is provided by a cam timer (R. S. Components, London). This has six switches and is arranged to operate at 2 r.p.m. Switch No. 1 is wired in series with the synchronous motor that drives the timer, so the motor stops once each cycle and can only be started again when switch 1 is shorted by the sampler as described above.

Switch 2 of the cam timer closes for 2 s and provides power for one of the two circuits of the valve motor, thus the valve is rotated to the sample position.

Switch 3 of the cam timer then closes for 5 s providing power to the pump which aspirates sample into the valve, and at the same time flushes out the preceding sample from the spectrophotometer.

Switch 4 then closes for 2 s and provides power for the second circuit of the valve motor which rotates the valve back to the delivery mode.

Switch 5 then closes briefly and initiates the operation of the precision timer. This is a semi-conductor precision timer ZN 1034E (R. S. Components, London); its circuit was assembled from details provided by the supplier. The precision timer switches on the pump motor for a precise time which is adjustable between 0·5 and 1000 s. The pump propels buffer substrate and the sample slug through the reactor tubing to the cuvette where it is arrested. After the sample slug is arrested in the cuvette, the trace is recorded for 105 s before the next cycle commences.

The general arrangement of the equipment, excluding the spectrophotometer, is shown in the photograph (figure 3).

The preliminary setting of the precision timer is achieved by pumping sodium tetraborate (0·1 mol/l), using 0·4% Bromothymol blue as sample and recording the absorbance at 600 nm. The recorder trace is examined, the timer adjusted and the cycle repeated until the recorder trace stops just at the top of the peak. It is convenient if the recorder chart drive can be reversed so that each trace can be superimposed on its predecessor.

Before evaluating the system, the efficiency of the arrangements for pre-heating the reaction mixture was tested by use of a thermochromic solution as suggested by Bowie *et al.* [9]. Operating at 30°C the mixture had reached 29·4°C within 10 s of arresting the pump and this proved to be a considerable improvement over the original manual technique.

### Evaluation of the system

As a preliminary evaluation the system was first used to measure albumin by the dye-binding method using bromocresol purple as proposed by Pinnell and Northam [10]. It was then used in the kinetic mode to measure lactate dehydrogenase (LDH). Finally, its ability to carry out the $\alpha_2$ macroglobulin ($\alpha_2$M) kinetic assays was evaluated. Details of the experimental conditions are as follows.

### *Reagents*

Unless otherwise stated all chemicals were obtained from BDH Ltd, Poole, Dorset, UK.

(1) Bromocresol purple (BCP) reagent for albumin determination: 0·1 g of BCP, 6 g of sodium acetate trihydrate,

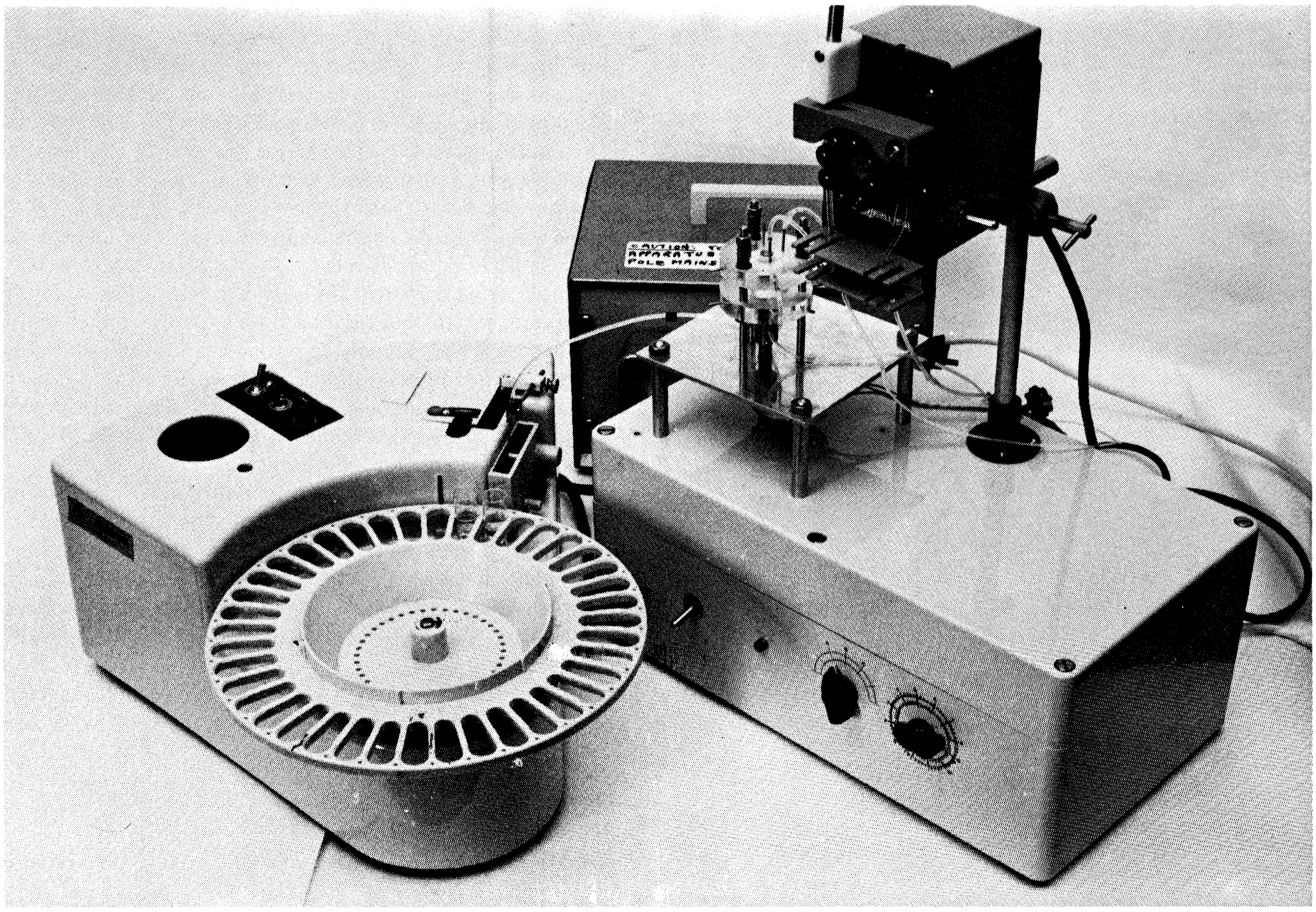

*Figure 3. Autosampler, valve and pump.*

4 ml of ethanol, 2 ml of glacial acetic acid and 4 ml of 30% w/v Brij 35 were dissolved in deionized water and made up to 1 l.

(2) Albumin solutions: human albumin fraction V (Sigma Co. Ltd, Poole, Dorset, UK) was dissolved in deionized water to give standards of 20, 30, 40, 50 and 60 g/l.

(3) LDH standards: the LDH assay was standardized with the aid of Ortho Normal Human Control Serum, lot No. W27XO2B (Ortho Diagnostics, High Wycombe, UK).

(4) Stock phosphate buffer (1 mol/l, pH 7·4): 1·36 g of potassium dihydrogen orthophosphate and 3·3 g of sodium hydroxide were dissolved in water and made up to 1 l.

(5) LDH reagent: the stock phosphate buffer was diluted 1 : 10 and 0·050 g of sodium pyruvate and 0·110 g of nicotinamide-adenine dinucleotide (reduced) disodium salt (NADH) added to each litre. This working reagent was prepared fresh daily.

(6) $\alpha_2$M reagents.

(*a*) Tris buffer pH 7·9-8·1: 86 ml of 1 mol/l Tris, 50 ml of 1 mol/l hydrochloric acid and 20 ml of 1 mol/l calcium chloride were made up to 1 litre with deionized water.

(*b*) Trypsin: bovine pancreatic trypsin (Boehringer Corporation, Lewes, Sussex, UK) was made up to 0·3 g/l in buffer (*a*).

(*c*) BAEE: N-α-benzoyl-L-arginine ethyl ester hydrochloride was made up to 0·17 g/l in buffer (*a*).

### *Albumin determination*

Working conditions were as follows. Albumin reagent was pumped as the carrier stream at 2 ml/min. The sample volume was 10 μl (determined by the size of the port in the valve). The reaction coil consisted of 2 m of 0·7 mm i.d. PTFE tubing. The dispersion, measured as described by Růžička and Hansen [5] and using a solution of BCP, was found to be 45. The machine was operated in an end-point mode, that is the pumping period was extended so that the slug of sample passed completely through the cuvette without halting and the absorbance at 620 nm was displayed as sharp peaks on the recorder. Sample concentration was calculated from the peak heights.

A range of albumin standards was examined and a plot of their absorbances was found to be linear over the range 20–60 g/l. An assortment of patient's sera were then examined. One serum, repeated 10 times, gave a coefficient of variation of 2% at a level of 42 g/l. Twenty-one sera were assayed by the flow-injection method and by a method employing Bromocresol green on the Vickers M300; the coefficient of correlation was 0·93.

### *Lactate dehydrogenase determination*

The conditions were as follows. The flow arrangements were exactly as in figure 1. The LDH reagent was pumped as the carrier liquid at 1 ml/min. The sample volume was 10 μl. The reaction coil was of PTFE, 350 mm long and 1·0 mm i.d. The dispersion of the system was 12. The total cycle time of 2 min was made up of 45 s pumping to transfer the sample to the cuvette, 15 s of 'settling' time and 60 s during which the plot of absorbance was recorded. The absorbance was measured at 340 nm and the operating temperature was 30°C. The slopes of the plots were measured with a protractor. A series of samples of patients' sera were examined by this technique. One sample with a level of 213 IU/l (normal range up to 195 IU/l) was repeated 10 times and gave a coefficient of variation of 4·8%. Twenty-two samples were assayed and the results compared with end-point assays obtained on the same samples with similar reagents with the Vickers M300. The coefficient of correlation was 0·99.

### *$\alpha_2$ macroglobulin kinetic studies*

The performance of the machine in measuring the kinetic behaviour of samples of human sera under the conditions prescribed by Topping and Seilman [1] was finally studied. A series of dilutions of serum in buffer were prepared to give a final volume of 0·4 ml for each dilution. (Trypsin solution: 0·1 ml was added to each and the mixture maintained at 30°C for 10 min.) In the manual technique, 0·1 ml of the mixture was added to 3·0 ml of BAEE and the absorbance of the final mixture at 253 nm recorded for 2 min.

The working conditions for the application of the technique to the new machine were as follows. The reaction coil consisted of 600 mm of 0·5 mm PTFE tubing. The sample volume was 10 $\mu$l and the dispersion was 26. The BAEE substrate was pumped at 1 ml/min.

A single serum sample was assayed 20 times to determine the precision of the system; the coefficient of variation was 4·5%. A collection of 77 assorted dilutions of several sera were assayed by both the manual and the automatic techniques. The coefficient of correlation was 0·986.

### Discussion

In assembling an FIA system the choice of pump is important. Peristaltic pumps are most commonly used and, by virtue of their design, inevitably produce pulsation. Closely spaced rollers increase the frequency and decrease the amplitude of the pulsation; a small level of pulsation is unimportant and may even be desirable [11]. The Ismatec mini S pump used in this assembly appears to be the only inexpensive pump available which meets this requirement. For stopped-flow operation it has the added advantage that it stops abruptly immediately its power supply is interrupted.

For stopped-flow work, the timing of the pumping phase which transfers the sample to the cuvette is critical. Electromechanical timers are generally insufficiently precise and too difficult to adjust for this purpose and it would thus be impracticable to make use of one switch of the cam timer. Fortunately, precise integrated circuit timers are inexpensive, are readily available and clearly serve the purpose well.

The system is remarkably stable, so that once the timer is adjusted to stop the pump with the sample slug centred in the cuvette, no further adjustment is needed for 2 h or more. At present, the timer is reset for each batch of studies but less frequent adjustment may be adequate.

The precision of the system appears to be adequate, since the figures for LDH compare favourably with those generally obtained with other methods in the authors' laboratory. Measurement of the slope of the plot by protractor must make a significant contribution to the error, and, no doubt, measurement of the slope electronically would be an improvement. The precision obtained with the albumin method was relatively poor, but the fixed volume of 10 $\mu$l of sample was much too large for the method.

The speed of the system is modest, so that the total time required for a complete $\alpha_2$M assay, using a combination of the dilutor and flow-injection system, is a little over half that required for a manual assay. However, it was the intention to relieve tedium and free the analyst, rather than to make a major increase in throughput, and this has been achieved.

The next step of the operation will be aimed at increased throughput. A design for a further machine is in preparation. This will have some of the features of the machine described by Snook *et al.* [12] and will monitor the reaction kinetics of 25 samples simultaneously, thus increasing the throughput by an order of magnitude or more.

### References

1. Topping, R. M. and Seilman, S., *Biochemical Journal*, **177** (1979), 493.
2. Riley, C., Watson, J. D. McK., Morgon, J., Rocks, B. F., Sheya, M., Oldfield, P. R. and Ewen, C., *Journal of Automatic Chemistry* **5** (1983), 27.
3. Růžička, J. and Hansen, E. H., *Analytica Chimica Acta*, **78** (1975), 145.
4. Stewart, K. K., Beecher, G. R. and Hare, P. E., *Analytical Biochemistry*, **70** (1976), 167.
5. Růžička, J. and Hansen, E. H., In *Flow Injection Analysis* (Wiley Interscience, New York, 1981), p. 104.
6. Mindegaard, J., *Analytica Chimica Acta*, **104** (1979), 185.
7. Anderson, L., *Analytica Chimica Acta*, **110** (1979), 123.
8. Růžička, J. and Hansen, E. H., *Analytica Chimica Acta*, **106** (1979), 207.
9. Bowie, L., Esters, F., Bolin, J. and Gochman, N., *Clinical Chemistry*, **22** (1976), 449.
10. Pinnell, A. E. and Northam, B. E., *Clinical Chemistry*, **24** (1978), 80.
11. Vanderslice, J. T., Stewart, K. K., Rosenfeld, A. G. and Higgs, D. J., *Talanta*, **28** (1981), 11.
12. Snook, M., Renshaw, A., Rideout, J. M., Wright, D. J., Baker, J. and Dickens, J., *Journal of Automatic Chemistry*, **1** (1979), 72.

